# Bariatric Surgery in Youth: the Perspective of Dutch Pediatricians, Parents, and Adolescents

**DOI:** 10.1007/s11695-021-05648-8

**Published:** 2021-08-06

**Authors:** Kelly G. H. van de Pas, Daniëlle S. Bonouvrie, Loes Janssen, Yvonne G. M. Roebroek, Bas S. H. J. Zegers, Wouter K. G. Leclercq, Anita C. E. Vreugdenhil, François M. H. van Dielen

**Affiliations:** 1 Department of Surgery, Máxima Medical Center, Veldhoven, The Netherlands; 2 Department of Pediatrics, Maastricht University Medical Center, Maastricht, The Netherlands; 3 NUTRIM School of Nutrition and Translational Research in Metabolism, Maastricht University, Maastricht, The Netherlands; 4 Department of Pediatrics, Máxima Medical Center, Veldhoven, The Netherlands

**Keywords:** Obesity, Bariatric surgery, Youth, Perspective

## Abstract

**Background:**

Recent studies have indicated that bariatric surgery is effective for the treatment of youth with severe obesity. The attitudes of pediatricians, parents, and adolescents regarding this topic remain unclear. Therefore, the aim of this study was to assess the current thoughts and beliefs of Dutch pediatricians, parents, and adolescents regarding bariatric surgery in youth.

**Methods:**

An online survey containing twenty questions on bariatric surgery in youth was distributed to pediatricians of the Dutch Society of Pediatrics. Parents and adolescents who participated in an interdisciplinary care program for overweight, obesity, and severe obesity filled out an online survey of twelve questions.

**Results:**

One hundred and twenty-one pediatricians, 49 parents, and 19 adolescents completed the surveys. Seventy-two pediatricians (59.5%) considered bariatric surgery to be an effective treatment for youth with severe obesity when conventional treatment fails, and intend to refer patients for bariatric surgery. The most frequently suggested conditions for bariatric surgery were a minimum age of 16 years (*n* = 59, 48.7%), a BMI threshold of 40 kg/m^2^ (*n* = 51, 42.2%), and a minimum Tanner stage of IV (*n* = 59, 48.8%).

Thirty parents (61.2%) and fourteen adolescents (73.7%) responded that bariatric surgery should become available for youth with severe obesity.

**Conclusion:**

Dutch pediatricians, parents, and adolescents increasingly accept bariatric surgery as a treatment modality in youth with severe obesity who do not respond successfully to lifestyle intervention. Whether pediatricians will actually refer youth for bariatric surgery remains to be seen when this treatment option will be implemented in the Netherlands.

**Graphical abstract:**

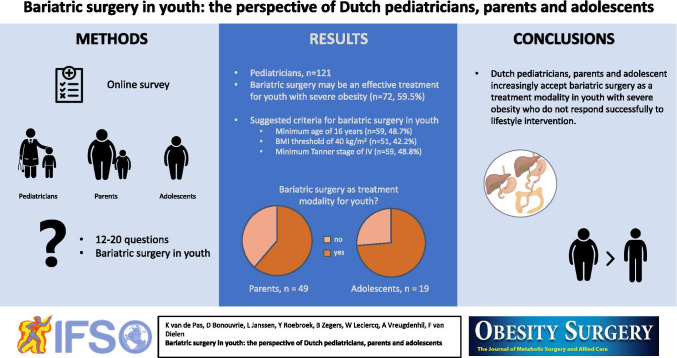

**Supplementary Information:**

The online version contains supplementary material available at 10.1007/s11695-021-05648-8.

## Introduction

The worldwide prevalence of overweight and obesity in youth has increased substantially in the last decades; in 2015 more than 100 million children and adolescents were obese [1–3]. Although the rising trends of overweight and obesity have plateaued, the rates of severe obesity are still growing with data from 2009 indicating that 0.59% of Dutch boys and 0.53% of Dutch girls were affected by severe obesity [4]. This upward trend is concerning when considering the substantial short- and long-term health risks related to severe obesity, such as type 2 diabetes mellitus (T2DM), hypertension, fatty liver disease, and dyslipidemia, even when compared to youth with obesity [5–9].

The standard treatment for youth with obesity in the Netherlands comprises of multimodal lifestyle intervention programs focusing on dietary behavior, physical activity, and underlying individual and systemic factors, provided by a pediatric multidisciplinary team. These programs have shown to result in a significant decrease in body mass index (BMI) and improvement of cardio-metabolic risk parameters in youth with overweight, obesity, and severe obesity [10–13]. At the same time, a quarter of treated youth do not experience weight loss and adolescents with severe obesity have proven to be particularly difficult to treat [11, 12].

As bariatric surgery is commonplace in the treatment of adult obesity, it can be considered in youth with severe obesity to achieve similar long-lasting weight loss and concurrent improvement of comorbidities when conventional treatment fails. A meta-analysis by Shoar et al. reported that bariatric surgery is safe and effective in the treatment of severe obesity in adolescents aged 12 to 19 years old [14]. However, long-term follow-up is lacking [14–16]. A recently published guideline for the treatment of youth with overweight and obesity in the Netherlands advised reticence towards bariatric surgery, advocating its use in youth only in the context of scientific research. Furthermore, this guideline stated that referral should be considered by pediatricians of obesity expertise centers and after the unsuccessful completion of at least 1 year of multidisciplinary lifestyle intervention at named centers. A successful intervention is defined as weight loss of ≥ 10% [17]. In line with the cautious approach of this guideline, the opinions of professionals, parents, and adolescents worldwide on this subject are divided [18–23]. Studies among pediatricians from the USA dating from 2007 to 2009 reported that 47.0% and 88.5% of pediatricians would not refer patients for bariatric surgery [19, 20]. Another qualitative report revealed that Dutch obesity specialists experience reluctance to refer youth for bariatric surgery as they endorse concerns that surgery might not treat the underlying psychological or behavioral problem. On the other hand, the obesity specialists, parents, and adolescents who felt that the etiology of obesity was predominantly somatic were more in favor of bariatric surgery [21]. However, the current perspective of Dutch pediatricians, parents, and adolescents remains unclear.

With the goal of further investigating the efficacy and feasibility of bariatric surgery in youth, our aim was to explore the current attitudes of Dutch pediatricians towards these topics [24]. A secondary aim was to discover the thoughts and beliefs of Dutch parents and adolescents regarding bariatric surgery in youth.

## Methods

### Study Design

In January 2020, an online survey was distributed to all practicing members of the Dutch Society of Pediatrics in the Netherlands. To optimize response rates, a reminder was sent to the pediatric departments of all Dutch hospitals from September to November 2020.

Adolescents (13–18 years) who were treated for their overweight, obesity, or severe obesity in the outpatient, family-based, interdisciplinary care program of the obesity expertise Centre for Overweight Adolescent and Children’s Healthcare (COACH) at the Maastricht University Medical Centre (MUMC +) were asked to fill out a survey during their follow-up visits from September to December 2020. Their parents, as well as parents to children under 13 years of age who were treated for their overweight, obesity, or severe obesity in the COACH program, were asked to fill out a survey in the same period [11]. To optimize response rates, an email was sent to distribute the survey to the parents and adolescents.

The study protocol was submitted to our local Medical Ethical Research Committee, who deemed formal approval not necessary according to Dutch law (Medical Research Involving Human Subjects Act).

### Survey

Anonymous surveys were designed using an online platform for questionnaires and surveys (Survey Monkey Inc., San Mateo, CA, USA) (Appendix 1). The surveys were self-administered and the study aim was explained before the start of all the surveys.

The survey for pediatricians consisted of 20 questions covering demographics, the current practice of youth with severe obesity including the results of this treatment, and the opinions of the respondents regarding bariatric surgery in youth. Youth was defined as persons aged < 18 years old, and severe obesity defined as a BMI ≥ 40 kg/m^2^ or a BMI ≥ 35 kg/m^2^ with an obesity-related co-morbidity, both adjusted for gender and age according to the International Obesity Task Force cut off points [25]. Regarding the questions on bariatric surgery in youth, the pediatricians had to assume that the youth followed a lifestyle intervention program for at least 12 months without successful weight loss, and that they had stable and supportive families.

The survey for parents and adolescents consisted of twelve questions covering their current treatment and their perspectives on bariatric surgery in youth. A short introduction was given to the parents and adolescents regarding bariatric surgery. Types of questions included dichotomous, multiple-choice, and Likert scale questions. In all surveys, some questions allowed textual remarks.

### Statistical Analysis

The sample size was based on the most important question; a dichotomous question regarding the willingness of pediatricians to refer for bariatric surgery. Accepting a maximal margin of error of 0.1 (precision) for proportions in our population of interest, we required a minimum sample size of 97 pediatricians to estimate proportions close to 0.5 with sufficient precision [26]. All completed surveys were used for analysis. Continuous data are presented as mean ± standard deviation (SD). Categorical data are presented as number (percentage). Statistical analysis was performed using IBM SPSS Statistics version 25 (IBM, Armonk, NY, USA).

## Results

The results of the pediatricians, parents, and adolescents are presented separately.

### Pediatricians

Of the 1461 pediatricians who are affiliated with the Dutch Society of Pediatrics, 176 (12.0%) filled in the questionnaire including 128 complete responses. After excluding the seven responses of pediatric residents, 121 responses were analyzed. Most of the pediatricians were general pediatricians, working in a non-academic hospital and currently treating 1–5 children for severe obesity (Table 1).

**Table 1:** Baseline characteristics of respondents and their practice. *n*, number

	Pediatricians
Number of complete responses — *n*	121
Years of working experience including residency (mean ± SD)	18.8 ± 8.2
Differentiation — *n* (%)	
General pediatrician Pediatric endocrinologist Pediatric gastro-enterologist Other	96 (79.3%) 2 (1.7%) 5 (4.1%) 18 (14.9%)
Hospital — *n* (%)	
Center of expertise for children with obesity Non-academic hospital Academic hospital Other	10 (8.3%) 95 (78.5%) 11 (9.1%) 5 (4.1%)
Children currently on treatment for severe obesity — *n* (%)	
None 1–5 children 6–15 children 16–30 children More than 30 children Other	16 (13.2%) 51 (42.2%) 18 (14.9%) 6 (5.0%) 16 (13.2%) 14 (11.6%)

#### Current Practice

**Fig. 1:**
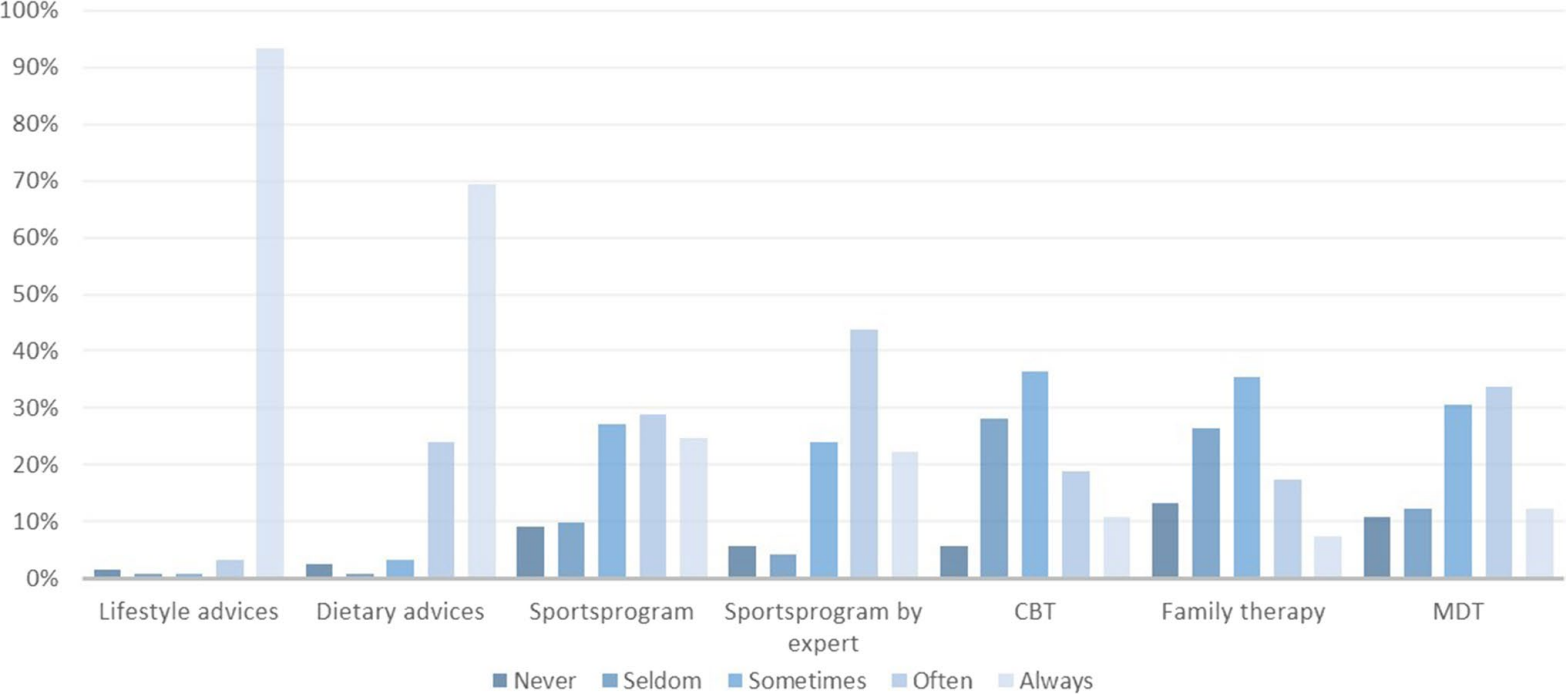
Reported frequency of providing different treatment modalities in youth with severe obesity. CBT, cognitive behavioral therapy; MDT, multidisciplinary treatment

One hundred and thirteen pediatricians (93.4%) reported that they always offered lifestyle advice to youth with severe obesity, and 84 pediatricians (69.4%) responded that they always referred to a dietician for dietary advice (Fig. 1).

Different norms of treatment success were observed; 54 respondents (44.6%) considered stabilization of bodyweight after 12 months of intervention as successful and 33 (27.3%) considered improvement of obesity-related comorbidities as a successful treatment, independent of bodyweight change. Twenty-six (21.5%) and 8 pediatricians (6.6%) reported that they considered a weight loss of respectively ≥ 5% or ≥ 10% after 12 months of intervention as successful. Ninety-three pediatricians (76.9%) estimated that ≤ 25% of the youth with severe obesity were treated successfully in their hospital. If their treatment was unsuccessful, referral to an obesity expertise center could be the “add on” treatment according to 56 pediatricians (46.3%). Eighteen pediatricians (14.9%) reported that they would refer for inpatient treatment, and ten (8.3%) for bariatric surgery, assuming this would be an option. Seven out of the ten pediatricians who would refer for bariatric surgery were working at a pediatric obesity expertise center.

#### Pediatricians’ Perspective on Bariatric Surgery in Youth

Seventy-two pediatricians (59.5%) shared the opinion that bariatric surgery may be effective in treating youth with severe obesity that were unsuccessfully treated with lifestyle interventions. These pediatricians would also refer for bariatric surgery. Eleven pediatricians (9.1%) did not believe bariatric surgery could be an effective treatment and 38 (31.4%) were inconclusive. Forty-nine pediatricians (40.5%) responded that they would not refer for bariatric surgery, with the reasons varying from “lack of evidence and experience” to “referral via an obesity expertise center.”

**Fig. 2:**
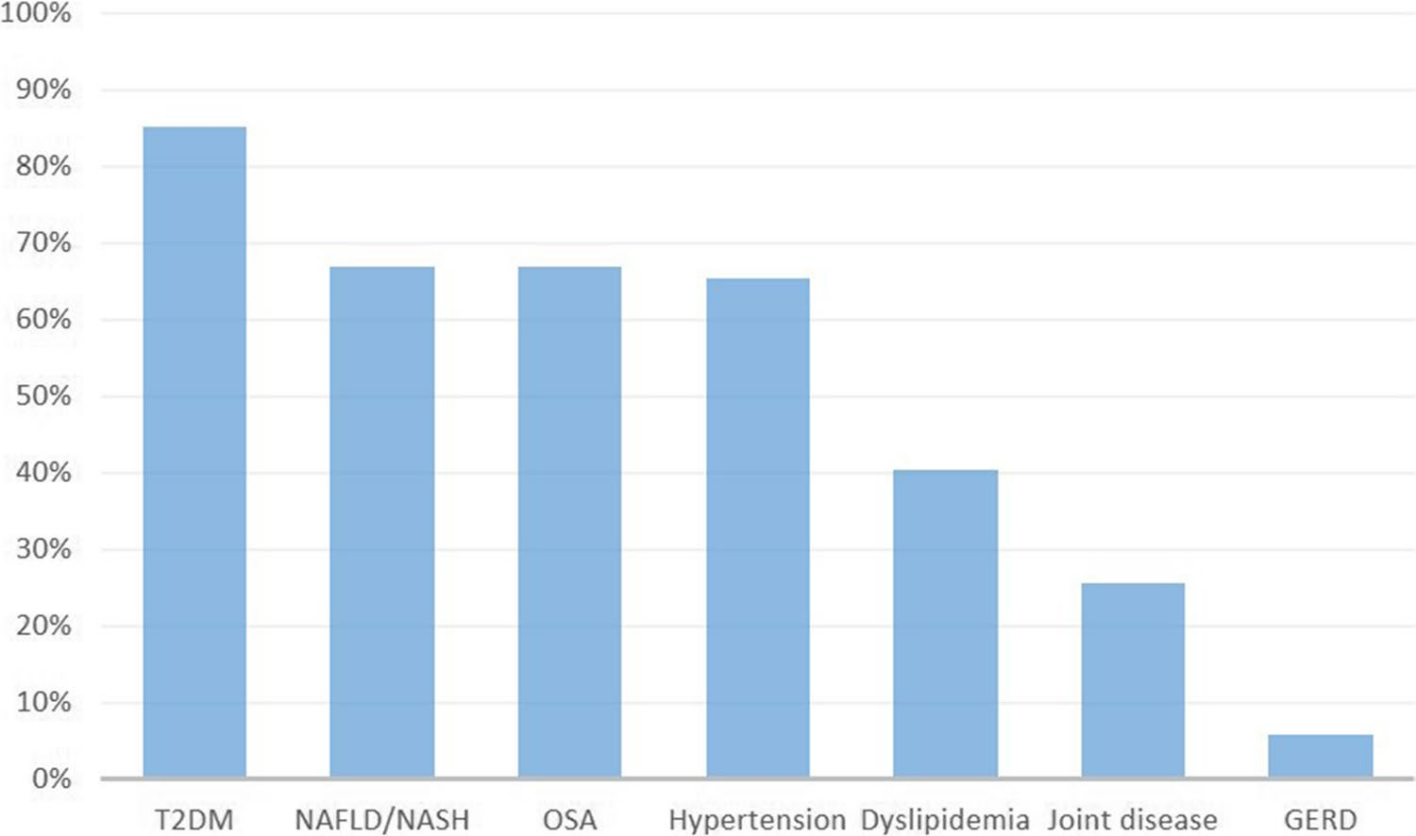
Comorbidities associated with a lower limit of BMI for bariatric surgery in youth. T2DM, type 2 diabetes mellitus; NAFLD/NASH, non-alcoholic fatty liver disease/non-alcoholic steatohepatitis; OSA, obstructive sleep apnea; GERD, gastroesophageal reflux disease

The majority (*n* = 113, 93.4%) of the respondents reported that there should be a minimum age for bariatric surgery in youth, with 59 pediatricians suggesting a minimum age of 16 years (48.7%). Regarding a BMI threshold for surgery, 51 pediatricians (42.2%) suggested a lower limit of 40 kg/m^2^ (sex and age adjusted) without comorbidities, whereas 38 respondents (31.4%) would prefer a BMI of 35 kg/m^2^ without comorbidities. When comorbidities are present, the BMI threshold declined for 106 respondents (87.6%). Most often, T2DM was chosen as an influential comorbidity, followed by non-alcoholic fatty liver disease/non-alcoholic steatohepatitis (NAFLD/NASH), obstructive sleep apnea (OSA), and hypertension (Fig. 2). Besides BMI and the presence of comorbidities, also physical development expressed by Tanner stage appeared to be of importance. According to 59 (48.8%) and 46 pediatricians (38.0%), a Tanner stage of IV or V respectively was the minimum for bariatric surgery in youth.

The majority of the respondents (*n* = 82, 67.7%) reported that bariatric surgery should become a common treatment modality for selected adolescents with severe obesity who do not benefit from lifestyle intervention. The most common reasons for reluctance were that “it should not become a common treatment modality, only a last resort treatment option” and “lack of evidence.”

### Parents of Youth with Overweight, Obesity, or Severe Obesity

Of the 159 parents whose children were treated at COACH and were approached, 56 (35.2%) filled in the questionnaire including 49 complete responses. The children of the respondents were affected by overweight, obesity, or severe obesity for at least 1 year, the majority for 3–5 years (*n* = 20, 40.8%) or 6–10 years (*n* = 18, 36.7%). Thirty-three (67.3%) of their children were treated for their overweight, obesity, or severe obesity at the COACH program for at least 1 year.

Thirty parents (61.2%) reported that bariatric surgery should be available for youth with severe obesity if lifestyle intervention is not successful, and 22 (44.9%) would allow their child to be referred for bariatric surgery if the current treatment fails. Reasons for not allowing their child to be referred varied from “being too young” to “children are still growing.” Twenty-six parents (53.1%) were in favor of a minimum age for bariatric surgery, with a minimum age of 16 and 18 years both answered most frequently (*n* = 8, 16.3%).

Twenty-nine parents (59.2%) responded that their child could decide to undergo bariatric surgery without the approval of their parents, after reaching the age of 16 or 17 years. Almost all respondents reported that bariatric surgery should be offered alongside a family-based program around the surgery (*n* = 45, 91.8%). The most frequently reported main goal for surgery was weight loss according to the parents (*n* = 20, 40.8%), followed by improvement of obesity-related comorbidities (*n* = 10, 20.4%) and self-esteem (*n* = 10, 20.4%).

### Adolescents with Overweight, Obesity, or Severe Obesity

Of the 30 adolescents who were treated at COACH and were approached, 19 (63.3%) completed the questionnaire. The adolescents had a mean age of 15.5 ± 1.6 years. All adolescents were affected by overweight, obesity, or severe obesity for at least 1 year, with a majority of twelve adolescents (63.2%) for 6 years or longer. Eleven (57.9%) of the adolescents were treated for their overweight, obesity, or severe obesity at the COACH program for at least 1 year.

Fourteen adolescents (73.7%) reported that bariatric surgery should be available, and 11 (61.1%) wanted to undergo bariatric surgery themselves if their current lifestyle intervention is not effective. Eight of the ten adolescents (80.0%) who were 16 years or older responded that they could make the decision for bariatric surgery independently of their parents. There was no consensus on the program around bariatric surgery in youth; 31.6% of the adolescents would prefer an individual program (*n* = 6), 36.8% a program with involvement of the parents (*n* = 7), and 31.6% a program with involvement of parents, brothers, and sisters (*n* = 6). The main goal of bariatric surgery reported by the adolescents was weight loss (*n* = 12, 63.2%), followed by improvement of self-esteem (*n* = 4, 21.0%) and improvement of obesity-related comorbidities (*n* = 3, 15.8%).

## Discussion

To the best of our knowledge, this is the first study in the Netherlands to have surveyed the current attitudes of pediatricians, parents, and adolescents towards bariatric surgery in youth. Our findings demonstrate that the majority of responding pediatricians consider bariatric surgery as a potentially effective treatment for youth with severe obesity. An even larger proportion agreed that it should be a common treatment modality for selected adolescents with severe obesity who are not responding to lifestyle interventions. Besides insufficient response to lifestyle interventions, pediatricians proposed a lower limit of BMI ≥ 40 kg/m^2^ (sex and age adjusted) without comorbidities, a minimum age of 16 years old, and a minimum Tanner stage of IV as criteria for bariatric surgery. This proposed minimum age criterion was comparable to the minimum age proposed by the parents.

Only a few studies have previously investigated the attitudes of pediatricians towards bariatric surgery in youth, revealing significant heterogeneity [19–21]. In 2009, an American report on pediatricians and family practitioners showed that 88.5% would be unlikely to, or would never refer a child for a bariatric procedure [19]. Conversely, another American study performed in 2007 with pediatricians reported that only 47.0% would decline referral for bariatric surgery [20]. In Europe, no studies have been conducted that have examined attitudes among pediatricians alone. However, a recent study among European pediatric surgeons has reported that 65.7% considered bariatric surgery to be a valuable contribution to obtain long-term weight loss in adolescents with severe obesity [27]. The findings in our study among pediatricians are most comparable to the European study among pediatric surgeons, revealing that 59.5% of the pediatricians would refer for bariatric surgery, and 67.7% supporting bariatric surgery as an acceptable treatment modality for a selected group of obese adolescents. We assume that that the accumulating evidence on safety and efficacy of bariatric surgery in youth explains why pediatricians are increasingly accepting this treatment modality [14–16].

Based on current American guidelines and Dutch guidelines, bariatric surgery in youth is accompanied by different selection criteria, including a lower limit of BMI [17, 28]. No age limit has been set in these selection criteria. Although, in different explorative studies, professionals have indicated their preference for a minimum age for bariatric surgery, yet this has ranged from 12 to 19 years old [18–20, 29, 30]. The proposed age of 16 years for bariatric surgery in our study is in line with the preferred age reported by a survey among members of the British Obesity and Metabolic Surgery Society and general practitioners [30]. Currently there is no evidence on limiting access to bariatric surgery in youth based on age [28]. The preference of professionals for a minimum age for bariatric surgery in youth could be due to less knowledge about the procedures and their consequences. Education for pediatricians who treat youth with severe obesity would therefore be recommended. Education of the pediatricians might lead to less of a barrier in referring youth for bariatric surgery, and eventually lead to better treatment of youth with severe obesity [31].

Until now, the thoughts and beliefs of parents and adolescents regarding bariatric surgery in youth have not been studied extensively [21–23, 32, 33]. A recent study by Singh et al. reported that 84.6% of parents would consider bariatric surgery after counselling by pediatricians, compared with only 34.5% of the parents without counselling [22]. In our study, 61.2% of parents stated that bariatric surgery should be available for youth with severe obesity, whereas only 44.9% of parents would allow their child to be referred for bariatric surgery if the current treatment was insufficiently effective. This discrepancy suggests that counselling by pediatricians could play a crucial role when discussing bariatric surgery in youth, which is supported by a qualitative study of parents and adolescents who underwent gastric banding [23].

Another important aspect of bariatric surgery in youth is family involvement, as concluded by Inge et al. in 2004, a motivated and supportive family is pivotal for successful bariatric surgery in youth [34]. This is in line with our findings that the majority of the parents and adolescents stated that bariatric surgery should only be offered with a perioperative family-based program.

A limitation of this study is the low response rate of the pediatricians. This might have led to selection bias in the results. To minimize this, the hospitals where the respondents worked at were compared with the hospitals where all the members of the Dutch Society of Pediatrics worked at, and the respondents more often worked at a non-academic hospital. Nevertheless, we still believe that this distribution of pediatricians across the Netherlands has provided an insight into their thoughts and beliefs regarding bariatric surgery in youth, as the pediatricians in non-academic hospitals are treating more youth with severe obesity compared to academic hospitals. Another potential limitation is that the parents and adolescents surveyed might not be a representative group, since they are being treated for their overweight, obesity, or severe obesity. Therefore, they may experience more positive attitudes regarding bariatric surgery in youth compared to the general population with overweight, obesity, or severe obesity. The third limitation is the small sample ﻿size﻿ and the limited response rate of the parents and adolescents. To minimize selection bias, the characteristics of the parents and adolescents were compared to the general COACH population, and they were comparable in terms of age and treatment duration.

## Conclusion

Dutch pediatricians increasingly accept bariatric surgery as a treatment modality in youth with severe obesity who do not respond successfully to lifestyle intervention, as long as conditions such as a minimum BMI, age, and Tanner stage are met. Whether pediatricians will actually refer for bariatric surgery remains to be seen when this treatment option will be implemented in the Netherlands.

## Supplementary Information

Below is the link to the electronic supplementary material.

Supplementary file1 (DOCX 23 KB)
