# Supplementary material for: Bariatric Surgery in Youth: the Perspective of Dutch Pediatricians, Parents, and Adolescents
Source: Obes Surg. 2021 Aug 6;31(11):4821–8. doi: 10.1007/s11695-021-05648-8 (PMC8490240; doi:10.1007/s11695-021-05648-8)
Supplement: Supplementary file 1 — Supplementary file1 (DOCX 23 KB) [file 11695_2021_5648_MOESM1_ESM.docx]

**Appendix 1 – Survey’s**

All surveys were administered in Dutch. Below is a translation of the questionnaires to English. This translation may lead to some nuanced differences.

# Pediatricians – Treatment of children with severe obesity

Through this survey we want to assess the current treatment of children with severe obesity in the Netherlands and the opinion of Dutch pediatricians on bariatric surgery as ‘add-on’ treatment to the current lifestyle intervention programs for children and adolescents with severe obesity.

**Demographic data**

1. What is your specialty?
2. General pediatrician
3. Pediatric endocrinologist
4. Pediatric gastroenterologist
5. Other, please provide further explanation __________________
6. What kind of hospital do you work in?
7. Expertise center for the treatment of children and adolescents with severe obesity
8. Non-academic hospital
9. Academic hospital
10. Other, please provide further explanation __________________
11. How many years have you worked as a pediatrician (*including pediatrician in training*)?

__________ years

**Current care and experience**

The following questions are asked to gain insight into the current treatment of children and adolescents with severe obesity and the results of this treatment.

A child is defined as a person with an age < 18 years. Severe obesity is defined as a BMI ≥ 40 kg/m^2^ or a BMI ≥ 35 kg/m^2^ with an obesity related co-morbidity, adjusted for gender and age according to the IOTF cut off points.

International IOTF Cut off points:

Boys: <http://s3-eu-west-1.amazonaws.com/wof-files/New_Cut_off_Points_Male_Children.pdf>

Girls: <http://s3-eu-west-1.amazonaws.com/wof-files/New_cut_off_points_female_children.pdf>

1. How many children with severe obesity do you currently treat in your hospital?
2. None
3. 1-5 children
4. 6-15 children
5. 16-30 children
6. > 30 children
7. Other, please provide further explanation __________________
8. Which forms of treatment do you use in your intervention program (never – seldom – sometimes – often – always)?
9. Lifestyle advice
10. Dietary advice provided by a dietician
11. Sports program not provided by a physiotherapist
12. Sports program provided by a physiotherapist
13. Cognitive behavioral therapy provided by a psychologist/behavioral therapist
14. Family therapy provided by a psychologist/pedagogue
15. Multidisciplinary lifestyle intervention program
16. Other, please provide further explanation __________________
17. When do you consider the provided treatment as successful?
    1. Weight stabilization after 12 months
    2. Weight loss ≥5% after 12 months
    3. Weight loss ≥10% after 12 months
    4. Improvement of obesity related comorbidities, independent of weight change
18. According to the definition you gave in the previous question, in how many children with severe obesity is the provided treatment effective?
19. ≤25%
20. >25-≤50%
21. >50-≤75%
22. >75%
23. What would be the best ‘add on’ treatment if the current treatment fails/is not effective (according to the definition you gave at question 6)?
24. Continue current treatment
25. Refer to primary care for lifestyle management
26. Refer to inpatient treatment
27. Refer to an expertise center
28. Refer for bariatric surgery (assuming that this is an actual option)
29. Other, please provide further explanation __________________
30. What percentage of the children with severe obesity without a successful treatment in your hospital will register for bariatric surgery in adulthood (18 years old)?
31. ≤25%
32. >25-≤50%
33. >50-≤75%
34. >75%
35. I don’t know
36. What percentage of the children with severe obesity with a successful treatment in your hospital will register for bariatric surgery in adulthood (18 years old)?
37. ≤25%
38. >25-≤50%
39. >50-≤75%
40. >75%
41. I don’t know
42. Is your treatment based on the policy described in the Section Guidelines: Obesity, guidelines for Pediatricians (2018)?
43. Yes
44. No
45. I don’t know

**Opinion bariatric surgery**

The following questions concern your attitude towards bariatric surgery in youth. In all questions you can assume that children followed a lifestyle intervention program for a minimum of 12 months without success and that the child has a stable and supportive family.

1. Do you believe that bariatric surgery can be an effective treatment for children with severe obesity who do not (sufficiently) respond to lifestyle intervention?
2. Yes
3. No
4. I don’t know
5. Would you refer a child with severe obesity who is not successful in the lifestyle intervention program for bariatric surgery?
6. Yes
7. No, explain why not: _______________
8. Do you think there should be a minimum age for bariatric surgery in youth?
9. Yes, specify the minimum age in years ____________
10. No
11. What would be the lower limit of BMI for bariatric surgery in youth (no comorbidities)?
12. 30 kg/m^2^
13. 35 kg/m^2^
14. 40 kg/m^2^
15. Other, specify the lower limit____________
16. Would the presence of a comorbidities influence the answer given at question 15?
17. Yes
18. No
19. Which of the following comorbidities would change the lower limit of BMI for bariatric surgery in youth (multiple answers possible)?
20. Type II diabetes Mellitus
21. Hypertension
22. Obstructive Sleep Apnea Syndrome
23. Dyslipidemia
24. Gastroesophageal reflux disease
25. Joint problems
26. Non-Alcoholic Fatty Liver Disease/ Non-Alcoholic Steatohepatitis
27. Other (please provide further explanation) __________________
28. What would be the minimum Tanner stage for bariatric surgery in youth (no comorbidities)?
29. Tanner stage I
30. Tanner stage II
31. Tanner stage III
32. Tanner stage IV
33. Tanner stage V
34. Do you believe that bariatric surgery should become a conventional treatment for selected adolescents with severe obesity who do not benefit from lifestyle intervention?
35. Yes
36. No, explain why not ________________________
37. If you have any comments or suggestions, please write them down below

# Adolescents – Treatment of children with severe obesity

Severe obesity in adults is treated with modifications in lifestyle (lifestyle intervention). Examples of lifestyle intervention are adjustments in eating patterns and exercise.

In a selected group of adults, in which the lifestyle intervention is not successful, weight loss surgery is performed. The two most commonly performed operations in the Netherlands are gastric sleeve, an operation in which the stomach is shortened lengthwise, and the gastric bypass, in which the stomach is reduced and the small intestine is bypassed.

For several years now these operations have been performed in children with severe obesity who do not respond to lifestyle intervention. Children lose about 20 to 30% of their total weight after weight loss surgery and additional diseases such as diabetes, high blood pressure and sleep apnea can be cured. Next to this, the quality of life of these children improves.

However, surgery involves risks. Complications that can occur in the short term are pneumonia, bleeding, wound infection and leakage of the bowels, and in the long-term gall stones, chronic abdominal pain and intestinal obstruction.

We want to investigate whether weight loss surgery is suitable for children, and therefore are curious about your opinion.

**Current treatment**

The following questions are asked to gain insight into your current treatment.

1. How long have you been overweight?
   1. <1 year
   2. 1-2 years
   3. 3-5 years
   4. 6-10 years
   5. >10 years
2. Which forms of treatment have you already had (never – seldom – sometimes – often – always)?
   1. Lifestyle advice
   2. Dietary advice provided by a dietician
   3. Sports program not provided by a physiotherapist
   4. Sports program provided by a physiotherapist
   5. Cognitive behavioral therapy provided by a psychologist/behavioral therapist
   6. Family therapy provided by a psychologist/pedagogue
   7. Multidisciplinary lifestyle intervention program
   8. Other, please provide further explanation __________________
3. How long have you been at COACH?
4. ≤6 months
5. 6 months – ≤1 year
6. 1 – ≤2 years
7. 2 – ≤3 years
8. 3 – ≤4 years
9. 4 – ≤5 years
10. > 5 years

**Opinion on weight-loss-surgery in children**

1. Do you believe that weight loss surgery should be available for children with severe obesity, in which multimodal lifestyle intervention is not successful?
   1. Yes
   2. No, why not____________
2. Do you think weight loss surgery in children with severe obesity should be an individual program for the child, or should the parents and possibly the brother(s) and/or sister(s) be involved?
   1. Individual program
   2. Program with parents
   3. Program with parents and brother(s)/sister(s)
3. Which weight loss surgery would you prefer?
   1. An operation that is reversible
   2. An operation that is NOT reversible
4. I don’t know
5. Which weight loss surgery would you prefer?
   1. Weight loss surgery with a lot of weight loss, but possibly more complications
   2. Weight loss surgery with slightly less weight loss, but less complications
   3. I don’t know
6. What would be your main goal in weight loss surgery?
   1. Weight loss
   2. Improvement of concomitant diseases (for example diabetes, high blood pressure, sleep apnea)
   3. Improving self-confidence/self-image
   4. Better functioning at school/work
   5. Other, please provide further explanation __________________
7. How old are you?
   1. 13 years
   2. 14 years
   3. 15 years
   4. 16 years
   5. 17 years
   6. 18 years
8. Do you want to undergo weight loss surgery, if the current treatment has no effect, before you turn 18 years old?
   1. Yes
   2. No, why not_____________

**16-17 years old**

Only complete the question if you are 16 or 17 years old.

1. Do you think you can make the choice to undergo weight loss surgery completely independent of your parents?
   1. Yes
   2. No, why not ____________
2. If you have any comments or suggestions, please write them down below.

# Parents - Treatment of children with severe obesity

Severe obesity in adults is treated with modifications in lifestyle (lifestyle intervention). Examples of lifestyle intervention are adjustments in eating patterns and exercise.

In a selected group of adults, in which the lifestyle intervention is not successful, weight loss surgery is performed. The two most performed operations in the Netherlands are gastric sleeve, an operation in which the stomach is shortened lengthwise, and the gastric bypass, in which the stomach is reduced and the small intestine is bypassed.

For several years now these operations are being performed in children with severe obesity who do not respond to lifestyle intervention. Children lose about 20 to 30% of their total weight after weight loss surgery and additional diseases such as diabetes, high blood pressure and sleep apnea can be cured. Next to this, the quality of life of these children improves.

However, a surgery involves risks. Complications that can occur in the short term are pneumonia, bleeding, wound infection and leakage of the bowels, and in the long-term gall stones, chronic abdominal pain and intestinal obstruction.

We want to investigate whether weight loss surgery is suitable for children, and therefore are curious about your opinion.

**Current treatment**

The following questions are asked to gain insight into the current treatment your child receives.

1. How long has your son/daughter been overweight?
2. <1 year
3. 1-2 years
4. 3-5 years
5. 6-10 years
6. >10 years
7. Which forms of treatment has your son/daughter already had (never – seldom – sometimes – often – always)?
   1. Lifestyle advice
   2. Dietary advice provided by a dietician
   3. Sports program not provided by a physiotherapist
   4. Sports program provided by a physiotherapist
   5. Cognitive behavioral therapy provided by a psychologist/behavioral therapist
   6. Family therapy provided by a psychologist/pedagogue
   7. Multidisciplinary lifestyle intervention program
   8. Other, please provide further explanation __________________
8. For how long have your son/daughter been treated at COACH?
9. ≤ 6 months
10. 6 months - ≤ 1 year
11. 1 year - ≤ 2 year
12. 2 year - ≤ 3 year
13. 3 year - ≤ 4 year
14. 4 year - ≤ 5 year
15. ≥ 5 year

**Opinions on weight loss surgery in children/adolescents**

1. Do you believe that weight loss surgery should be available for children with severe obesity, in which multimodal lifestyle intervention was not successful?
2. Yes
3. No, why not _____________
4. Would you allow your son/daughter to be referred for weight loss surgery, if the current treatment has no effect?
5. Yes
6. No, why not _____________
7. At what age would you let your son/daughter undergo weight loss surgery?
8. <10
9. 10 years
10. 11 years
11. 12 years
12. 13 years
13. 14 years
14. 15 years
15. 16 years
16. 17 years
17. ≥ 18 years
18. I would not let my son/daughter undergo weight loss surgery
19. Age is not an issue for me
20. Do you think that your son/daughter, if he/she is 16-17 years old, can decide on whether he/she should undergo weight-loss-surgery (without your input and approval)?
21. Yes
22. No, why not _________________
23. Should weight loss surgery in children with severe obesity be an individual program or should the family be involved?
24. Individual program
25. Family program
26. Which weight loss surgery would you prefer?
27. An operation that is reversible
28. An operation that is NOT reversible
29. I don’t know
30. Which weight loss surgery would have your preference?
31. Weight-loss-surgery with a lot of weight loss, but possibly more complications
32. Weight loss surgery with slightly less weight loss, but also less complications
33. I don’t know
34. What would be the main goal for you and your son/daughter in weight loss surgery?
35. Weight loss
36. Improvement of concomitant diseases (for example using less medication)
37. Improving self-confidence/self-image
38. Better functioning at school/work
39. Other, please provide further explanation __________________

12. If you have any comments or suggestions, please write them down below
